# Human Monocyte-Derived Suppressor Cell Supernatant Induces Immunoregulatory Effects and Mitigates xenoGvHD

**DOI:** 10.3389/fimmu.2022.827712

**Published:** 2022-03-08

**Authors:** Claire Gérard, Marine Thébault, Baptiste Lamarthée, Coraline Genet, Florine Cattin, Andréa Brazdova, Nona Janikashvili, Claudie Cladière, Marion Ciudad, Séthi Ouandji, Thibault Ghesquière, Hélène Greigert, Claire Tinel, Olivier Adotevi, Philippe Saas, Maxime Samson, Sylvain Audia, Bernard Bonnotte

**Affiliations:** ^1^ Université Bourgogne Franche-Comté (UBFC), Inserm, EFS BFC, UMR1098, Team « immunoregulation, immunopathology », RIGHT Interactions Greffon-Hôte-Tumeur/Ingénierie Cellulaire et Génique, Dijon, France; ^2^ Department of Internal Medicine, Dijon University Hospital, Dijon, France; ^3^ Institute of Organic Chemistry and Biochemistry of the Czech Academy of Sciences, Prague, Czechia; ^4^ Department of Immunology, Faculty of Medicine, Tbilisi State Medical University (TSMU), Tbilisi, Georgia; ^5^ UBFC, Inserm, EFS BFC, UMR1098, RIGHT Interactions Greffon-Hôte-Tumeur/Ingénierie Cellulaire et Génique, Besançon, France

**Keywords:** myeloid-derived suppressor cell, xeno GVHD, supernatant characteristics, GMP-good manufacturing practice, immunoregualtion

## Abstract

Recently developed cell-based therapies have shown potential for graft-versus-host disease (GvHD) mitigation. Our team previously developed a protocol to generate human monocyte-derived suppressor Cells (HuMoSC), a subpopulation of CD33+ suppressor cells of monocytic origin. CD33+HuMoSC successfully reduced xenoGvHD severity in NOD/SCID/IL-2Rγc^-/-^ (NSG) mice. While CD33+ HuMoSC culture supernatant inhibits T cell activation and proliferation, the recovery of CD33+ HuMoSC immunosuppressive cells and the subsequent production of their supernatant is limited. An attractive solution would be to use both the CD33+ and the large number of CD14+ cells derived from our protocol. Here, we assessed the immunoregulatory properties of the CD14+HuMoSC supernatant and demonstrated that it inhibited both CD4 and CD8 T cell proliferation and decreased CD8 cytotoxicity. *In vivo*, injection of CD14+HuMoSC supernatant reduced xenoGvHD in NSG mice. Furthermore, CD14+HuMoSC supernatant maintained its immunoregulatory properties in an inflammatory environment. Proteomic and multiplex analyses revealed the presence of immunosuppressive proteins such as GPNMB, galectin-3 and IL-1R(A) Finally, CD14+HuMoSC supernatant can be produced using good manufacturing practices and be used as complement to current immunosuppressive drugs. CD14+HuMoSC supernatant is thus a promising therapy for preventing GvHD.

## Introduction

Allogeneic hematopoietic cell transplantation (allo-HCT) is the only curative treatment for hematologic malignancies but it is limited by mortality and morbidity secondary to graft-versus-host disease (GvHD). The most common treatment for GvHD is immunosuppressive therapy, which prevents the proliferation of pathogenic T cells. The main drawback of this treatment is general immunosuppression, which leaves the patient vulnerable to infection and increases the risk of relapses. There is thus an unmet need for novel therapies. Myeloid-derived suppressor cells are innate immune cells that acquire the capacity to suppress adaptive immune responses (1, 2). In the context of allo-HCT, these cells may mediate immune suppression through multiple mechanisms (3). Our team has previously described an original approach to obtaining human myeloid suppressor cells derived from circulating monocytes (patent N°WO2015140077A1). These CD33-positive immunosuppressive cells were named human monocyte-derived suppressor cells (HuMoSC) (4, 5). We showed that CD33+HuMoSC are able to efficiently inhibit T cell proliferation in a STAT3-dependent manner. In addition, they induce regulatory T cells (Treg) population and significantly prevent GvHD in a xenogeneic murine model without impairing graft-versus-leukemia effect (5). Moreover, CD33+HuMoSC did not required cell-to-cell contact to limit T cell proliferation and their culture supernatant induced a very strong anti-proliferative effect (4). However, after culture, the fraction of CD33+HuMoSC generated with our protocol was relatively limited. In the present study, we hypothesized that in addition to CD33+, the more numerous CD14+ cells (CD14+HuMoSC) generated by our protocol could be harvested to produce a culture supernatant with high immunosuppressive properties. We thus assessed the potential impact of CD14+HuMoSC culture supernatant on effector CD4+ and CD8+ T cells, the major cell populations involved in the physiopathology of GvHD. In addition, we evaluated the efficiency of CD14+HuMoSC supernatant on GvHD prevention in a clinically relevant model of GvHD using NOD/SCID/IL-2Rγc-/- (NSG). Regarding its future therapeutic use, we demonstrated that CD14+HuMoSC supernatant can be used in an inflammatory environment or in combination with several drugs, and can be produced according to good manufacturing practices (GMP). Furthermore, proteomic and multiplex analyses revealed the presence of immunosuppressive proteins such as GPNMB, galectin-3 and IL-1RA, which could play a role in the CD14+HuMoSC supernatant effect. Altogether, these results suggest that CD14+HuMoSC supernatant could be a new therapeutic opportunity to prevent GvHD.

## Materials and Methods

### Generation of CD14+HuMoSC

CD33+HuMoSC generation has been described previously (4, 5). Briefly, peripheral blood mononuclear cells (PBMC) were isolated from buffy coats of healthy donors by Ficoll density gradient centrifugation. Monocytes were purified from PBMC by Percoll density gradient centrifugation. CD33+HuMoSC and CD14+HuMoSC were generated by incubating monocytes (2.10^6^ cells/mL) in X-vivo 15™ (Lonza, Switzerland) supplemented with recombinant human GM-CSF (10 ng/mL) and IL-6 (10 ng/mL) (both from Miltenyi Biotec, Paris, France) for 7 days. The unselected cultured cells were harvested and cryopreserved in X-vivo 15™ supplemented with 10% DMSO (controlled rate freezing of 1°C/min) at -80°C. CD33+HuMoSC and CD14+HuMoSC were obtained after CD33+ or CD14+ selection, respectively, from fresh or cryopreserved unselected cultured cells.

### Magnetic Cell Sorting

CD33+ HuMoSC, CD14+HuMoSC and T cells were purified by magnetic cell sorting using human CD33+, human CD14+ and Pan T cell isolation kits, respectively (Miltenyi Biotec). Magnetic separation was performed using an autoMACS-ProTM separator according to the manufacturers’ instructions (Miltenyi Biotec).

### Culture Supernatant Production

For CD14+HuMoSC supernatant, CD14+HuMoSC were generated as previously described and after sorting, CD14+HuMoSC were plated in physiological saline (Sodium Chloride 0.9%, Baxter, France) at 1.10^6^ cells/mL for the indicated time at 37°C in 24-well plates. As CD14+HuMoSC are semi adherent cells, supernatant was then delicately collected by pipetting and transferred in 15mL conical sterile tubes. In order to remove potential cell contaminant, supernatant was centrifuged at 1500 rpm for 5 minutes and carefully collected by pipetting. For production of monocyte supernatant, cells obtained after Percoll density gradient were plated in physiological saline in the same conditions as HuMoSC before supernatant collection. As the negative control, an equivalent volume of pure physiological saline (vehicle) was used.

### T Cell Proliferation and Suppression Assays

PBMC and T cells were stained using Cell Trace Violet according to the manufacturer’s procedure (Cell Trace™, Invitrogen, Cergy Pontoise, France). Labeled cells were cultured with Human T-activator CD3/CD28 beads (Dynabeads, Invitrogen) in a 24-well plate in 500µL of complete RPMI at 1.10^6^ cells/mL with or without 500µL of CD14+HuMoSC supernatant. For dose-dependent experiments, the volume of complete RPMI (500µL) and concentration of labeled cells (1.10^6^ PBMC or T cells/mL) was fixed whereas the volume of supernatant varied from 500µL (ratio 1:1), 250µL (ratio 1:2) or 0µL (ratio 0). For 1:2 and 0 ratios, 250µL or 500µL of physiological saline were added respectively. T cell division was detected after 5 days by flow cytometry using a LSRII cytometer (BD Biosciences) and analyzed using ModFit^®^ software (version 5.0). The proliferation index (*Pi*) measured by the ModFit software for both negative control (Unstimulated PBMC treated with physiological saline) and positive control (PBMC stimulated with anti CD3/CD28 and physiological saline) were used in the following equation to obtain the percentage of inhibition of CD14+ HuMoSC supernatant:


$$Percentageofinhibition=100\times \frac{\left(Pi_{StimVehicle}{-}_{}Pi{}_{UnstimVehicle})-(Pi_{StimCD14HuMoSCsup}{-}_{}Pi_{UnstimVehicle}\right)}{\left(Pi_{StimVehicle}{-}_{}Pi_{UnstimVehicle}\right)}$$

CD14+HuMoSC supernatant suppressive activity was evaluated in PBMC through the presence or absence of pro-inflammatory cytokines (human recombinant IL-2, IFN-γ, TNF-α, IL-1β, Miltenyi Biotec) at a concentration of 100 µg/mL or TLR ligands (LPS, Pam3Cys-SK4, Poly I:C, Flagellin, either, *In vivo*gen, Miltenyi or Fisher Scientific) at a concentration of 50 ng/mL. In indicated experiments, anti-inflammatory and immunosuppressive agents, such as methylprednisolone (MP, 25 ng/mL), methotrexate (MTX, 2.5 ng/mL) and cyclosporine (CsA, 5 ng/mL) were added to stimulated PBMC. These drug concentrations have been previously determined to induce a limited inhibition of PBMC proliferation (<50%). The additive immunosuppressive effect of CD14+HuMoSC supernatant can thus be measured in the presence of a given drug.

### Antibodies and Flow Cytometry Analysis

Flow cytometry analyses were performed as previously reported using monoclonal antibodies against CD3 (Biolegend, Ozyme, Saint-Cyr-l’Ecole, France, clone OKT3), CD4 (Miltenyi Biotec, clone REA623), CD8 (Biolegend, clone SK1), CD25 (Biolegend, clone BC96), IL-4 (BD Biosciences, Grenoble, France clone OKT3), IL-17A (Biolegend, clone BL168) and IFN-γ (eBioscience,ThermoFisher, Paris, France, clone 45.B3). Treg were stained with anti-Foxp3 (Alexa 488) (Human Treg FlowTM Kit, Biolegend, clone 259D). For discrimination of viable cells, cells were stained with Fixable Viability Stain (BD Biosciences) following manufacturer instructions. Cells were analyzed using a LSRII cytometer (BD Biosciences) and data analysis was performed with the FlowJo^®^ software (BD Biosciences, version 10.0.7r2).

### Xenogeneic Model of GvHD

Eight to 12 week-old NOD.Cg-Prkdcscid Il2rgtm1WjI/SzJ (NSG) mice (male and female, Charles River, Ecully, France) were subjected to 2Gy total body irradiation (TBI) at day 0 and were transplanted intraperitoneally (I.P.) with 10×10^6^ human PBMC. They were then treated with CD14+HuMoSC supernatant or physiological saline (vehicle) (2mL per mouse I.P.) once a week as depicted in **Figure 5A** Mice were scored twice a week until day 60 in a blinded fashion for clinical signs of GvHD (weight loss, general appearance of the fur, and mobility). Incidence of GvHD, clinical score and survival was noted for each mouse. Mice were euthanized when the clinical endpoints were reached (>15% weight loss, hunched posture, ruffled fur, reduced mobility, tachypnea). Blood analyses were performed from 150-200 µL of heparinized blood withdraw from mandibular or tail vein.

### Proteomic Analysis

CD14+HuMoSC supernatant protein composition was compared to monocyte supernatant produced as previously described. Samples were frozen at -20°C and transferred to the Plateforme d’Analyse Protéomique de Paris Sud-Ouest (PAPPSO). Samples were concentrated on mass filter of 3 kDa and then migrated on a gel. The gel bands were then digested and LC–MS/MS analyses of protein digests (400 ng of peptides) were performed using a NanoLC-Ultra System (nano2DUltra, Eksi-gent, Les Ulis, France) connected to a Q-Exactive mass spectrometer (Thermo Electron, Waltham, MA, USA). Peptide and protein identification were performed with X!Tandem software (6) (version Alanine 2017.2.01), using the human genome (uniprotKb_H_sapiens_181105) and a homemade contaminant database. Protein inference was performed using X!TandemPipeline (7) with the following parameters: at least two peptides per protein, peptide e-value <0.01, protein e-value <10−5. The false discovery rate (FDR) was assessed by searching a decoy database and estimated at 0.57% for peptides and 0% for proteins. Proteins represented by at least two reproducible and consistent peptides were quantified by summing their intensities, as in Balliau et al. (8), in order to measure their relative abundance. Only proteins free from contamination by bovine serum albumin were considered for analysis. These proteomic data were publicly deposited (https://doi.org/10.6084/m9.figshare.17128733.v1).

For subcellular location, the UniProtKB database was interrogated (uniprot.org).

### Multiplex Analysis

Concentrations of IFN-γ, IL-1RA, GPNMB, LEG3, Granzyme B and IL-17A were determined in cell-culture supernatant by using the Luminex Assay according to the manufacturer’s instructions (Multiplex kit,R&D Systems).

### Statistics

To analyze statistical significance between two groups, a non-parametric Mann-Whitney test was used. Overall survival was calculated as the time from the day of injection of PBMC until death. Data were censored after 60 days of follow-up. Kaplan-Meier curves were plotted and differences were evaluated using the log-rank test. For proteomic analysis, an analysis of variance using a linear model was used as well as a *post-hoc* Tukey analysis in order to highlight proteins with significant variations. Radar plot, alluvial plot, principal component analysis (PCA) and heatmap were obtained using R software (version 4.1.1) and the following packages: fmsb_0.7.1, networkD3_0.4, heatmap3_1.1.9, FactoMineR_2.4 and factoextra_1.0.7, respectively. For other statistical analyses, Prism 5 software was used (GraphPad Software) and a 2-tailed *P* value ≤ 0.05 was considered significant.

### Study Approval

The animal protocol (reference #15834) was approved by Institutional Animal Care and Use Committee of the Université de Bourgogne. The human study was approved by the Etablissement Français du Sang (EFS: ENR-B1-051, Besancon, France) and informed consent was obtained in compliance with the Declaration of Helsinki.

## Results

### CD14+HuMoSC Produce an Immunosuppressive Supernatant

**Figure 1:**
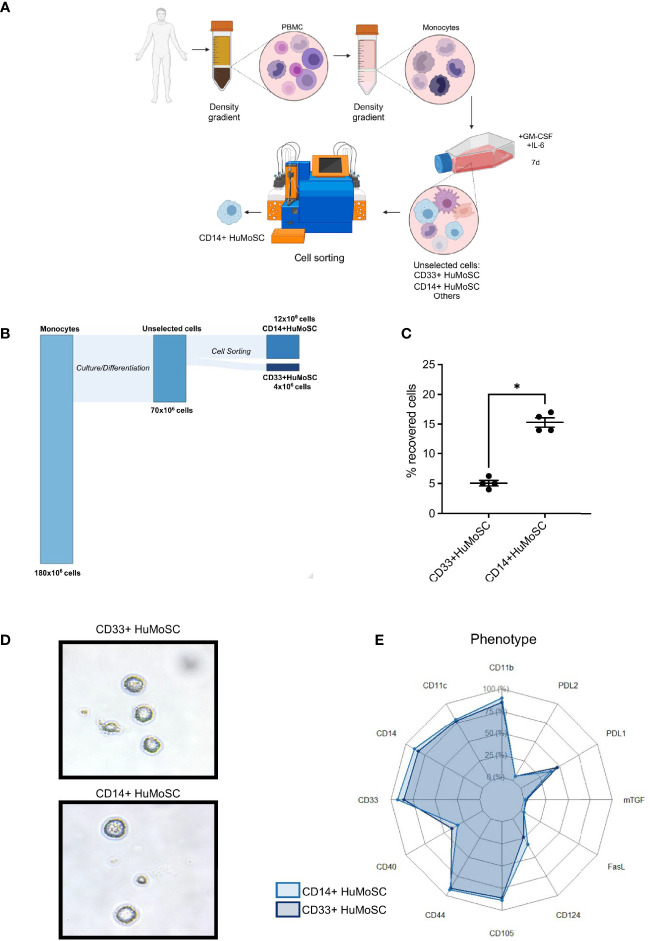
Protocol for CD14+HuMoSC generation. **(A)** Schematic workflow of both CD33+HuMoSC and CD14+HuMoSC generation. **(B)** Alluvial plot illustrating the proportion of harvested cells at each step of the protocol. **(C)** Following HuMoSC generation, the percentage of recovered cells was evaluated after selection for both CD33+HuMoSC and CD14+HuMoSC fractions. Results of 4 independent experiments are shown. **(D)** Representative morphology of both CD14+HuMoSC and CD33+HuMoSC characterized by optical microscopy (magnification x500). **(E)** Phenotypes of CD14+HuMoSC and CD33+HuMoSC were determined by flow cytometry. Results of 6 independent experiments are shown. Data are shown as mean ± SEM of representative experiments. Two-tailed Mann Whitney test: *p < 0.05.

**Figure 2:**
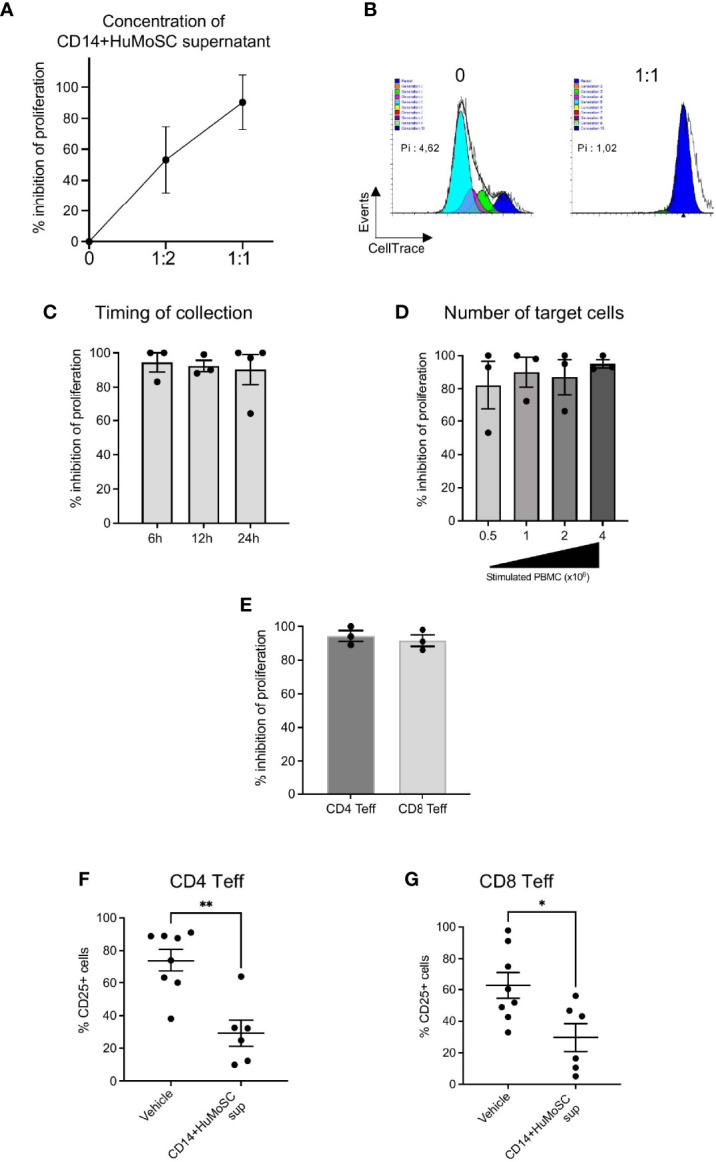
Characterization of optimal production parameters to produce an immunosuppressive supernatant derived from CD14+HuMoSC. **(A)** Concentration: Cell-Trace Violet labeled PBMC stimulated with anti-CD3/CD28 microbeads were cultured in presence of CD14+HuMoSC supernatant. Three different ratios of supernatant volume were assessed- 0, 1:1 and 1:2 volume of supernatant/volume of culture medium. The proliferation index was determined after 4 days and the percentage of proliferation inhibition was calculated. Results of 3 independent experiments are shown. **(B)** Representative Modfit analysis is shown. Pi, proliferation index; %: inhibition of proliferation. **(C)** Time of collection: CD14+HuMoSC were plated for 6h, 12h and 24h at concentration of 1 million cells/mL in physiological saline. At the indicated time point, Cell-Trace Violet labeled PBMC stimulated with anti-CD3/CD28 microbeads were cultured in presence of CD14+HuMoSC supernatant. The proliferation index was determined after 4 days and the percentage of proliferation inhibition was calculated. Results of 3 independent experiments are shown. **(D)** 500 000, 1 million, 2 million and 4 million of PBMC stimulated by anti-CD3/CD28 microbeads for 4 days were treated with CD14+HuMoSC supernatant, and PBMC proliferation was analyzed. Results of 3 independent experiments. **(E)** PBMC were stimulated with anti-CD3/CD28 microbeads for 4 days with or without CD14+HuMoSC supernatant Percentage of T cell proliferation inhibition was determined separately for CD4+ and CD8+ T lymphocytes by flow cytometry. Results of 4 independent experiments are shown. **(F, G)**. CD25 expression on CD4+ and CD8+ T cells stimulated by anti-CD3/CD28 microbeads for 4 days with or without CD14+HuMoSC supernatant. Results of 6 independent experiments. Data are shown as mean ± SEM of representative experiments. Two-tailed Mann Whitney test: *p < 0.05, **p < 0.01.

We previously showed that CD33+HuMoSC efficiently inhibited T cell proliferation and induced Treg differentiation (5), independent of cell-to-cell contact. In fact, CD33+HuMoSC culture supernatant induced very strong anti-proliferative effect (4). However, the number of CD33+HuMoSC generated with our protocol was relatively limited (**Figures 1A, B**). In contrast, the number of CD14+ cells retrieved after HuMoSC differentiation and cell sorting was three times higher than for CD33+HuMoSC (4.4% of CD33+HuMoSC vs 15.31% of CD14+ HuMoSC, p=0.0286, **Figure 1C**). In term of morphology, the two cell types were alike: they were large and semi-adherent cells presenting short dendrites (**Figure 1D**). Their phenotypes were also very similar, including elevated expression of CD11b, CD11c, CD44 and CD105 and intermediate expression of CD40, PD-L1 and CD124. In contrast, mTGF, FasL and PD-L2 expression were very limited in both cells (**Figure 1E**). Considering that CD33+HuMoSC immunosuppressive properties were previously reported (4, 5), we were interested in determining whether CD14+HuMoSC supernatant could provide similar effects. In this aim, total PBMC activated by CD3/CD28 beads were cultured in medium supplemented with increasing volumes of CD14+HuMoSC supernatant for five days. We observed that CD14+HuMoSC supernatant inhibited PBMC proliferation in a dose-dependent manner: one volume of supernatant mixed with one volume of medium inhibited proliferation by more than 90% (**Figures 2A, B**). Moreover, we tested whether the time of supernatant production by CD14+HuMoSC was a determining factor. Supernatant, collected after either 6h, 12h or 24h of CD14+HuMoSC plating in physiological saline, showed a massive inhibition of proliferation, irrespective of collection time (**Figure 2C**). We thus chose to collect the supernatant after 24h of plating and to use a volume ratio of 1:1 in all further experiments. Interestingly, the inhibitory function did not gradually decline with the number of target cells, seeing as the same volume of CD14+HuMoSC supernatant comparably inhibited cell proliferation from 500 000 stimulated PBMC up to 4 million cells (**Figure 2D**). Next, we examined whether the impact of CD14+HuMoSC supernatant was similar in CD4+ and CD8+ effector T cells. As illustrated in **Figures 2E, F**, the inhibition of proliferation was equivalent in both cell types. Regarding T cell subsets, CD14+HuMoSC supernatant decreased both CD4 and CD8 effector T cell activation as depicted by the decrease of CD25+ fraction (**Figures 2F, G**). Moreover, we verified that the decrease in T cell proliferation was not due to a potential higher rate of T cell death (data not shown). Overall, these experiments showed that the higher yield of CD14+HuMoSC recovery after cell sorting makes it possible to produce more cell supernatant as compared to CD33+HuMoSC. The immunosuppressive effects of CD14+HuMoSC supernatant were as potent as CD33+HuMoSC for the inhibition of both CD4 and CD8 effector T cell proliferation.

### CD14+HuMoSC Supernatant Impairs Th1 Differentiation, CD8 Cytotoxicity and Is Associated With Both CD4 and CD8 Tregs Higher Proportions

**Figure 3:**
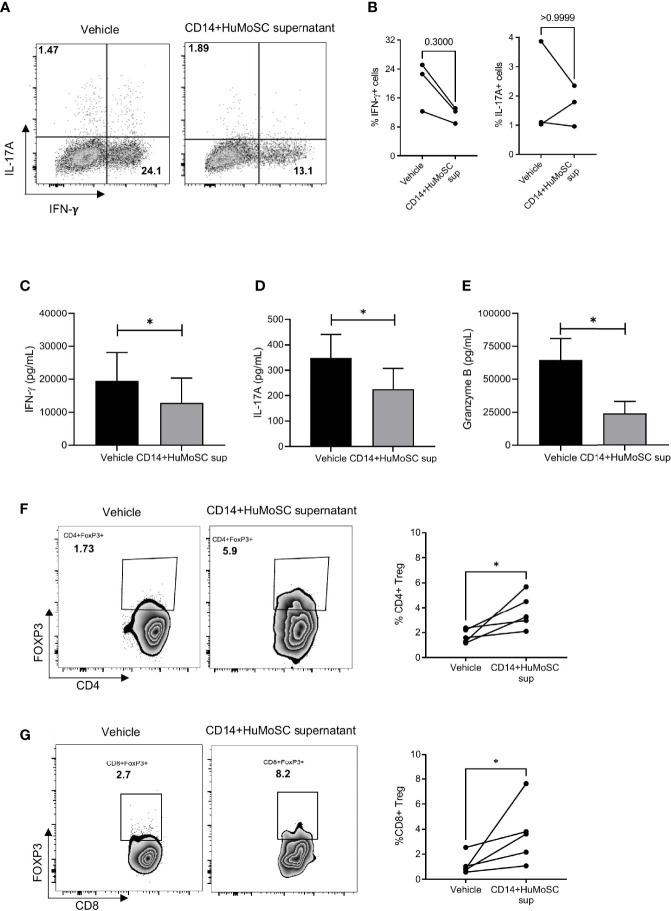
Effect of CD14+HuMoSC supernatant on T cell polarization. **(A-B)** After 4 days of stimulation with anti-CD3/CD28 stimulation beads and incubation with either CD14+HuMoSC supernatant or physiological saline (vehicle), T cells were stimulated for 4h with PMA+ionomycine and brefeldin A and then stained for IFN-γ, IL-4 and IL-17(A) Cytokine detection among CD4+ cells was performed by flow cytometry with BD LSR II and FlowJo software. **(A)** Dot plot from one representative experiment. **(B)** Percentage of IFN-g+ and IL-17A+ cells were calculated. Results of 3 independent experiments are shown. **(C–E)**. IFN-γ, Granzyme B and IL-17A concentrations were measured in the culture of stimulated T cells treated with or without CD14+HuMoSC supernatant. Results of 3 independent experiments are shown. **(F, G)**. Induction of CD4 and CD8 Treg with CD14+HuMoSC supernatant. Left panels: representative dot plots. Right panels: results of 6 independent experiments are shown. Data are shown as mean ± SEM of representative experiments. Two-tailed Mann Whitney test *p ≤ 0.05.

CD14+HuMoSC supernatant can affect T cell biology in multiple ways. We first studied whether CD14+HuMoSC supernatant could affect T cell polarization. In this aim, CD3+ purified T cells were subjected to antiCD3/CD28 stimulation in the presence of CD14+HuMoSC supernatant. Cytokine secretion was then assessed by flow cytometry and Luminex Assay. We observed that CD14+HuMoSC supernatant slightly decreased the percentage of IFN-γ-producing CD4+ T cells whereas the percentage of IL-17A producing cells was not altered (**Figures 3A, B**). The same type of analyses was also performed on CD8+ T cell polarization and showed that CD14+HuMoSC supernatant significantly increased the Tc2 population, leading to a significant decrease in the Tc1/Tc2 ratio (**Supplementary Figure 1A**). Cytokine measurement in the culture supernatant confirmed the total decrease in IFN-γ and IL-17A production whereas IL-4 levels were not perturbed (**Figures 3C, D** and **Supplementary Figure 1B**). We next assessed T cell cytotoxicity and we observed that CD14+HuMoSC supernatant decreased granzyme levels in the culture of stimulated effector T cells (**Figure 3E**). These results showed that CD14+HuMoSC supernatant treatment reduces IFN-γ, Granzyme B and IL-17A production by effector T cells. In addition, our team previously reported that CD33+HuMoSC induced Treg population in CD8 T cells (4). We therefore speculated that CD14+HuMoSC supernatant could also be associated with higher proportion of CD4 and CD8 Treg. Strikingly, the fraction of both CD4 and CD8 Treg was more than doubled after culture with CD14+HuMoSC supernatant for 4 days (**Figures 3F, G**). Altogether, these experiments demonstrated that CD14+HuMoSC supernatant strongly limits pathogenic type II IFN and IL-17A responses and tips the balance in favor of both CD4+ and CD8+Treg.

### CD14+HuMoSC Supernatant Sustained Immunosuppressive Properties in Inflammatory Context

**Figure 4:**
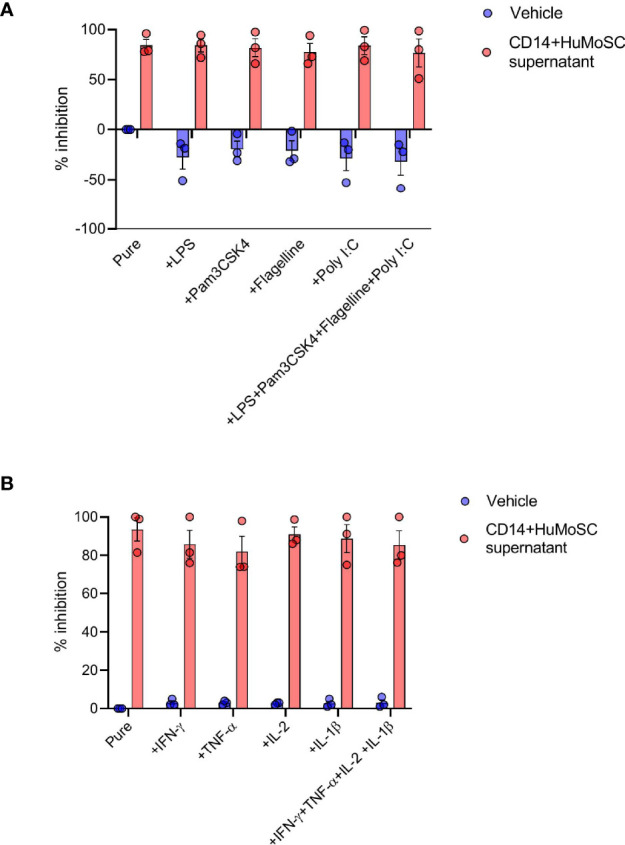
Inflammatory environment does not dampen CD14+HuMoSC supernatant immunosuppressive functions. **(A)** 500 000 Cell-Trace Violet labeled PBMC stimulated with anti-CD3/CD28 microbeads for 4 days were co-cultured in presence of LPS (50ng/mL), PAM3CSK4 (50ng/mL), flagellin (50ng/mL), Poly I:C (50µg/mL) or a combination of them and CD14+HuMoSC supernatant. PBMC proliferation is shown in 3 independent experiments. **(B)** 500 000 Cell-Trace Violet labeled PBMC stimulated with anti-CD3/CD28 microbeads for 4 days were co-cultured in presence of IL-2 (20IU/mL), IFN-γ (25ng/mL), TNF-α (25ng/mL), IL-1β (25ng/mL) or a combination of them and CD14+HuMoSC supernatant. PBMC proliferation is shown in 3 independent experiments. Data are shown as mean ± SEM of representative experiments.

Acute GVHD is the result of donor-derived alloreactive effector T cells attacking host tissues, including, but not limited to, the skin, liver and gut (9). In particular, epithelial damage due to the conditioning regimen compromises the intestinal barrier and facilitates pathogenic bacterial translocation (10). Major outer membrane components released by translocated bacteria are then recognized by the pathogen recognition receptor (PRR) of recipient antigen-presenting cell (APC), triggering alloreactive T cell proliferation (11). In order to evaluate the potential interest of CD14+HuMoSC supernatant in GvHD, we thus stimulated PBMC with antiCD3/28 beads supplemented with different PRR ligands such as LPS, Pam3CSK4, Flagelline or Poly I:C, and incubated the cells with or without CD14+HuMoSC supernatant for 5 days. When we measured PBMC proliferation, we observed that PRR ligands did not overcome the immunoregulatory properties of CD14+HuMoSC supernatant (**Figures 4A**). Moreover, many of the proinflammatory cytokines secreted following APC activation, such as IL-1β, TNF-*α*, IL-2 and IFN-*γ*, have been associated with GvHD pathophysiology and are implicated in the “cytokine storm” (12–14). We thus evaluated the capacity of CD14+HuMoSC supernatant to inhibit PBMC proliferation in presence of these cytokines. Remarkably, the immunosuppressive properties of CD14+HuMoSC supernatant were sustained in the presence of these cytokines both separately and in combination (**Figure 4B**). These results illustrated that the inflammatory context does not diminish the properties of CD14+HuMoSC supernatant.

### CD14+HuMoSC Supernatant Mitigates xenoGvHD

**Figure 5:**
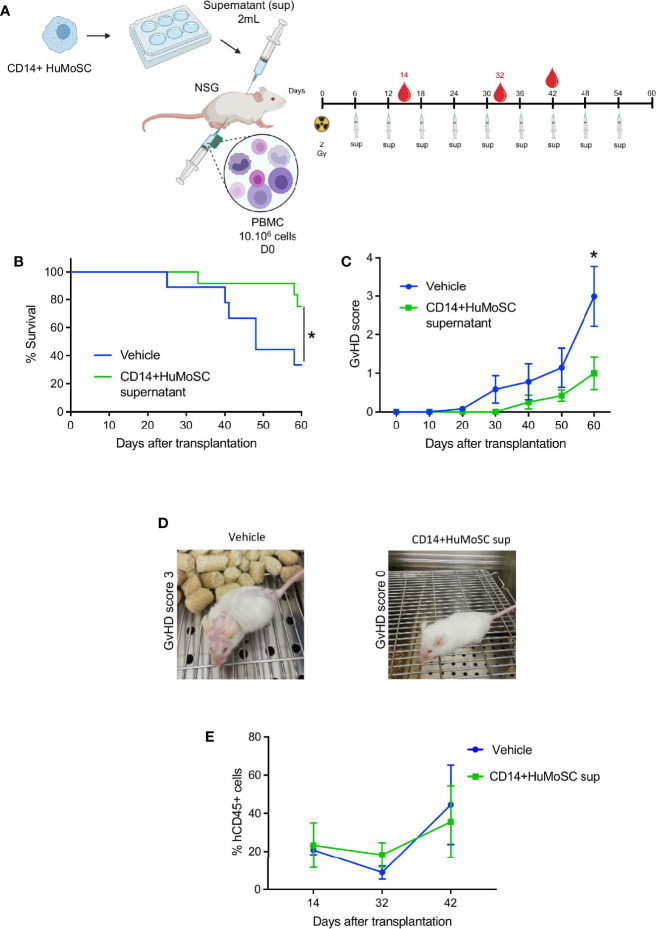
CD14+HuMoSC supernatant mitigated xenoGvHD in NSG mice. **(A)** Schematic representation of xenoGVHD model and treatments. NSG mice aged 8-12 weeks were irradiated (2 Gy) and received 10.106 huPBMC intraperitoneously and were then treated with 2 mL of physiological saline (vehicle, control group) or 2 mL of CD14+HuMoSC supernatant (CD14+HuMoSC supernatant group) once a week. At the indicated time, blood was harvested and human chimerism was evaluated by flow cytometry. **(B)** Survival curves were compared by using log-rank test. *p ≤ 0.05. **(C)** The GvHD score of the 2 groups was assessed twice a week. Two-tailed Mann Whitney test *p ≤ 0.05. **(D)** Photograph of two representative mice at day 60, a mouse treated with vehicle had a GvHD score of 3, a mouse treated with CD14+HuMoSC supernatant had a score of 0. **(E)** Human engraftment was evaluated at the indicated times using anti-human CD45 and anti-mouse CD45 antibodies. Results of 2 independent experiments and 9 mice per group are shown.

To assess the potential suppressive capacities of CD14+HuMoSC supernatant *in vivo*, we used a clinically relevant xenogeneic GvHD model based on the transfer of PBMC into TBI-conditioned NSG mice (**Figure 5A**). In this model, the onset of GvHD symptoms (weight loss, hunched posture, ruffled fur, reduced mobility, skin integrity and diarrhea) occurred after 30 days, while the GvHD clinical score peaked at 60 days. Regarding survival, two-thirds of mice transferred with PBMC and treated with physiological saline (vehicle) died within 60 days, as expected, whereas CD14+HuMoSC supernatant produced significant protection against xenoGvHD and related death (median survival: 48 days for vehicle vs undefined for CD14+HuMoSC supernatant, p = 0.047) (**Figure 5B**). The CD14+HuMoSC supernatant also significantly ameliorated GvHD scores on D60 (p=0.0247) (**Figures 5C, D**). Human chimerism was evaluated between day 14 and dy 42 and showed no differences between groups (**Figure 5E**), suggesting that CD14+HuMoSC supernatant did not limit engraftment but attenuated T cell xenoreactivity.

### CD14+HuMoSC Supernatant Can Be Produced According to GMP and Used as a Complement to Currently Prescribed Drugs

**Figure 6:**
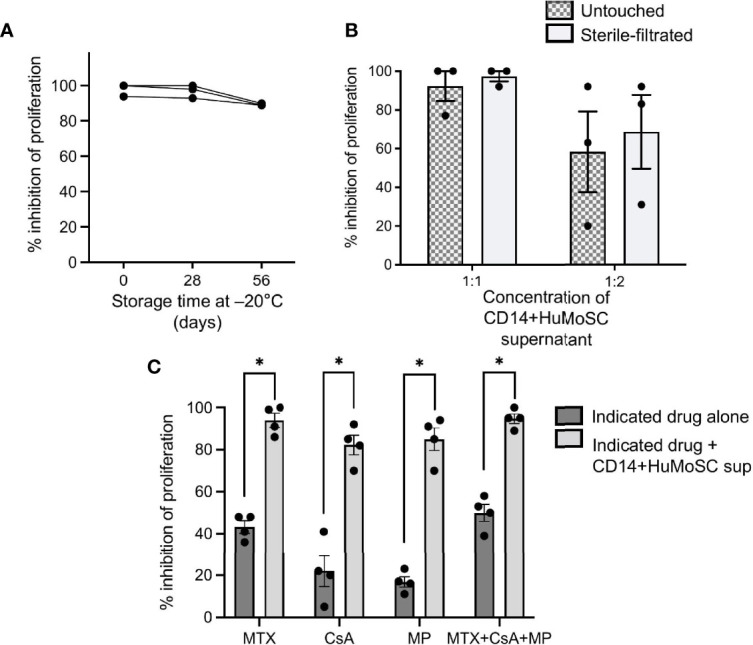
Production of CD14+HuMoSC supernatant for clinical use. **(A)** PBMC stimulated by anti-CD3/CD28 microbeads were co-cultured for 4 days with fresh or thawed CD14+HuMoSC supernatant after a storage at -20°C for 26 or 56 days, and their proliferation was analyzed by flow cytometry with BD LSR II and FlowJo software. **(B)** PBMC stimulated by anti-CD3/CD28 microbeads were co-cultured for 4 days with CD14+HuMoSC supernatant (volume sup/medium =1:1 or 1:2) recovered before and after filtration and their proliferation was analyzed by flow cytometry with BD LSR II and FlowJo software. Data for 3 independent experiments are shown. Data are shown as mean ± SEM of representative experiments. **(C)** 500 000 Cell-Trace Violet labeled PBMC stimulated with anti-CD3/CD28 microbeads were co-cultured in the presence of methotrexate (MTX, 2.5ng/mL), ciclosporin A (CsA, 5ng/mL), methylprednisolone (MP, 25ng/mL) or a combination of them with or without CD14+HuMoSC supernatant. Results for the inhibition of PBMC proliferation are shown for 3 independent experiments. Data are shown as mean ± SEM of representative experiments. Two-tailed Mann Whitney test *p ≤ 0.05.

Given the strong potential of CD14+HuMoSC supernatant as a cell-derived therapy product, we tried to approach our protocol of CD14+HuMoSC generation under GMP required conditions. First, we used using GMP-grade CD14 microbeads for CD14+HuMoSC isolation. Second, CD14+HuMoSC supernatant was produced in physiological (saline) solution, a clinically relevant medium that is usually used for drug administration. We then examined whether the freezing for 2 months at -20°C and sterilization with 0.22µm filters did not attenuate the immunosuppressive functions of CD14+HuMoSC supernatant. As depicted in **Figures 6A, B**, long-term frozen and sterile-filtrated CD14+HuMoSC supernatant was still able to strongly diminish PBMC proliferation. We then evaluated the potential of CD14+HuMoSC supernatant as a complement to the therapies that are currently prescribed in patients undergoing allo-HCT. The immunosuppressive function of CD14+HuMoSC supernatant was assessed in combination with immunosuppressive drugs usually prescribed for GvHD prevention such as methotrexate (MTX), cyclosporine A (CsA) and methylprednisolone (MP). As shown in **Figure 6C**, the addition of a limited concentration of immunosuppressive drugs in the culture induced a modest inhibition of the proliferation of stimulated PBMC, as expected. Strikingly, in combination with CD14+HuMoSC supernatant, proliferation was completely abrogated. Overall, these results indicated that CD14+HuMoSC supernatant can be produced on a large scale using GMP, and that it can be used in combination with existing treatments.

### CD14+HuMoSC Secretes LEG3, IL-1RA and GPNMB, Three Immunomodulatory Molecules

**Figure 7:**
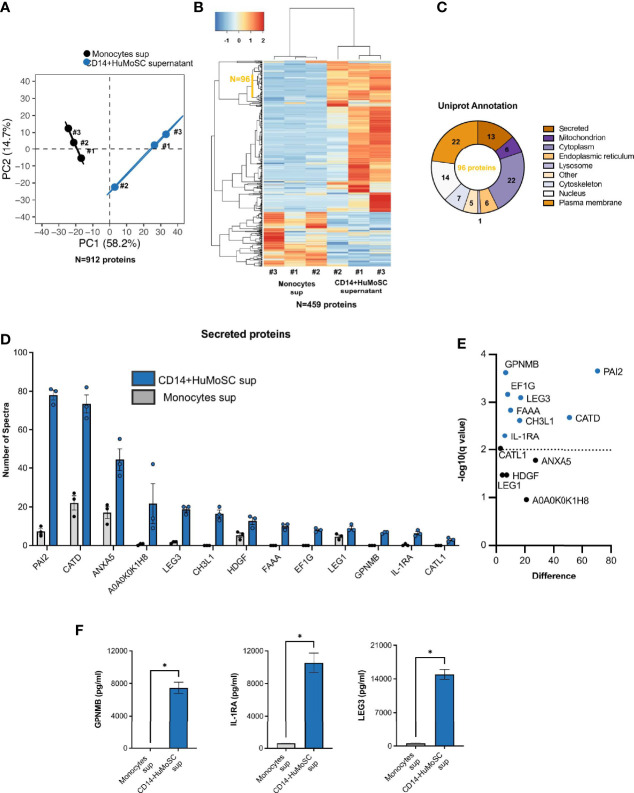
Proteomic analysis of CD14+HuMoSC supernatant composition compared with monocyte supernatant. Supernatants were produced by either undifferentiated monocytes or CD14+HuMoSC from 3 healthy volunteers and proteomic analysis was performed in order to characterize CD14+HuMoSC secretome. **(A)** Principal Component Analysis performed on 912 individual proteins. **(B)** Unsupervised heatmap of differentially secreted proteins revealed 96 consistently oversecreted proteins in CD14+HuMoSC supernatant. **(C)** Uniprot annotation revealed subcellular location of each of the 96 CD14+HuMoSC-specific proteins and revealed 13 secreted proteins. **(D, E)** Relative quantification of the 13 CD14+HuMoSC-specific secreted proteins with significant variation. **(F)** Confirmation of the composition of CD14+HuMoSC supernatant using Luminex Asssay. Comparaison of CD14+HuMoSC and monocyte (control) supernatants for IL-1RA, GPNMB and Galectin-3 secretions. Data for 3 independent experiments are shown. Data are shown as means ± SEM. Two-tailed Mann-Whitney test *p < 0.05.

In order to characterize the biological effectors that compose CD14+ HuMoSC supernatant and are responsible for its immuno-suppressive properties, we compared the protein composition of supernatant from fresh monocytes or CD14+ HuMoSC. In this aim, CD14+HuMoSC or fresh monocytes from 3 different donors were plated in physiological saline to produce supernatant and proteomic analysis was performed in order to identify the secreted proteins involved in immunosuppression. We retrieved 2508 groups of common peptides corresponding to 912 individual proteins. PCA based on information from these 912 quantified proteins strongly differentiated the two types of supernatants (**Figure 7A**), suggesting that CD14+HuMoSC secretomes diverge considerably from undifferentiated monocytes. Variance analyses identified 459 proteins differentially expressed in the two types of supernatant. Unsupervised hierarchical clustering confirmed the homogeneity in term of protein composition across replicates and highlighted the differences comparing cell types (**Figure 7B**). We noticed a cluster of 96 proteins consistently increased across CD14+HuMoSC supernatants. Interrogating online available database, 13/96 proteins were annotated as secreted proteins (**Figure 7C**). We then assessed the quantity of each of these proteins of interest, revealing significant increases in PAI2, GPNMB, LEG3, FAAA, CATD, EF1G, CH3L1 and IL-1RA in CD14+HuMoSC supernatants (**Figures 7D, E**). Using another platform of protein quantification by Luminex assay, we confirmed that CD14+ HuMoSC secreted high levels of IL-1RA, LEG3 and GPNMB compared to monocytes ([IL-1RA]= 10549 +/- 2381 pg/mL vs 606+/-72 pg/mL; [LEG3]= 14951 +/- 281 pg/mL vs 469+/-71 pg/mL; [GPNMB]= 7465 +/- 1358 pg/mL vs 0+/-0 pg/mL respectively) (**Figure 7F**). There was no commercially available kit to quantify the other candidates with this technique. Altogether, we identified three proteins, IL-1RA, GPNMB and LEG3, with potential immuno-suppressive properties in the CD14+HuMoSC supernatant.

## Discussion

Our team has previously reported an original approach for generating CD33+ human myeloid suppressor cells derived from circulating monocytes, named HuMoSC. However, the potential use of CD33+HuMoSC in a clinical context was hampered by the low yield of generated CD33+ HuMoSC. Herein, we modified our protocol in order to recover all of the CD14+ cells rather than just the CD33+ cells at the end of the culture. With this adjustment, we were able to obtain 3 times more cells, referred to as CD14+HuMoSC. When we analyzed the properties of the supernatant of these cells, we identified strong immunosuppressive capacities. In this work, we clearly demonstrated that CD14+HuMoSC supernatant modulated immune response on several levels, with marked effects on T cell proliferation and polarization. *In vitro*, CD14+HuMoSC supernatant inhibited effector T cell activation and was associated with a higher Treg proportion. These results may reflect that CD14+HuMoSC supernatant directly induces Tregs differentiation or that Tregs are less sensitive to CD14+HuMoSC supernatant treatment in the context of strong TCR stimulation. More experiments are needed to address this question.

*In vivo*, CD14+HuMoSC supernatant mitigated xenoGvHD, a severe inflammatory disease and preliminary results suggest that CD14+HuMoSC supernatant could decrease Tc1 commitment in CD8 as well as tissue lesions caused by cytotoxic T cells during xeno GvHD (data not shown) which are in line with *in vitro* observations but they need to be further confirmed. In addition, we showed that the combination of CD14+HuMoSC supernatant with immunosuppressive drugs usually prescribed for GvHD prevention such as MTX, CsA and MP increased the inhibition of T cell proliferation. These drugs can also interfere with T cell polarization, cytotoxicity and Treg commitment and thus the potential impact of combination with CD14+HuMoSC supernatant on these different outcomes is still needed. Moreover, given that these immunosuppressive drugs mitigate GvHD by themselves, we can speculate that their combination with CD14+HuMoSC supernatant would also diminish GvHD severity but more *in vivo* experiments are required to confirm this hypothesis. Proteomic analysis of supernatant composition identified new immunosuppressive candidates overexpressed in CD14+HuMoSC supernatant. Among these candidates, GPNMB, LEG3 and IL-1RA were confirmed by multiplex analysis. The presence of these immunosuppressive proteins could be the cause of the immunoregulatory properties of the CD14+HuMoSC supernatant, but further experiments are needed to confirm this hypothesis. Surprisingly, none of the well-characterized immunosuppressive factors expressed by MDSC such as CCL-2, IL-10, PD-L1, IL-2, IFN-α or IL-6 were detected in the CD14+HuMoSC supernatant.

In contrast, proteomic analysis and multiplex analysis showed that CD14+HuMoSC supernatant contains high levels of LEG3, GPNMB and IL1RA. LEG3 is an immunoregulatory protein also called galectin-3 and secreted by mesenchymal stem cells (15, 16), can affect both the immunological synapse between DC and T cells but also directly T cells by decreasing intracellular signaling and activation (17, 18). Similar to CD14+HuMoSC supernatant, galectin-3 has been described as causing Th1/Th2 imbalance in favor of the Th2 response (19). Although galectin-3 impairs T-cell function, it could also increase tumor cell proliferation and migration (20, 21). Thus, the high level of galectin-3 present in CD14+HuMoSC supernatant might increase possible recurrence rate after xenoGvHD treatment and could limit the benefit of CD14+HuMoSC supernatant. GPNMB is a transmembrane glycoprotein expressed on the surface of several cell types such as dendritic cells, macrophages, melanocytes, osteoblasts and Mo-MDSCs (22, 23). This protein binds syndecan-IV expressed on T cell surface and inhibits their proliferation. Interestingly, GPNMB has also been found in the extracellular environment. It has been shown that GPNMB can be secreted by IFN-γ/LPS-activated macrophages and that it plays a negative feedback role in inflammation (24). IL-1RA is the IL-1 receptor antagonist, which binds to the receptor and competitively inhibits the binding of IL-1β, preventing it from exerting its inflammatory effects. While IL-1RA is commonly used to slow the progression of moderate to severe active rheumatoid arthritis in patients (25), it shows promising results in other syndromes such as Familial Mediterranean fever, tumor necrosis factor associated periodic syndrome or acute gout (26–28). Very large doses are necessary to successfully block the effects of IL-1, probably due to the large number of IL-1 receptors present on the cell surface (25). Of course, the role of those three proteins in the immunomodulatory properties of CD14+HuMoSC supernatant need to be assessed by complementary experiments.

Finally, and perhaps more importantly, we demonstrated that a weekly injection of CD14+HuMoSC supernatant improved both survival and GvHD score in NSG mice. To our knowledge, this is the first time that an immunosuppressive supernatant from myeloid cells was shown to attenuate GvHD. In addition, patients undergoing allo-HCT can develop inflammatory conditions that influence suppressive cell therapies. Interestingly, neither PRR ligands nor inflammatory cytokines altered the immunosuppressive functions of CD14+HuMoSC supernatant on their own. This new therapeutic product, which could potentially be used to prevent GvHD, can be easily produced using GMP and combined with current medicines. Of course, the large-scale production of CD14+ HuMoSC supernatant using GMP-grade was not conducted in our study and the assessment of the feasibility of such production is still required.

In conclusion, we reported the clinical interest of a highly immunosuppressive supernatant derived from myeloid cells. Even though the precise mechanisms are not yet fully deciphered, we showed that CD14+HuMoSC supernatant inhibits CD4 and CD8 T cell activation, proliferation and cytotoxicity. Furthermore, CD14+HuMoSC supernatant attenuated xenoGvHD in NSG mice and sustained its immunosuppressive properties in inflammatory environment. These results pave the way for a new efficient therapeutic tool for GvHD.

## Data Availability Statement

The original contributions presented in the study are publicly available. This data can be found here: https://figshare.com/articles/dataset/20181105_bonnotte_Proteomic_Resultsfordeposition_xlsx/17128733/1.

## Ethics Statement

The studies involving human participants were reviewed and approved by Etablissement Français du Sang. The patients/participants provided their written informed consent to participate in this study. The animal study was reviewed and approved by Institutional Animal Care and Use Committee of the Université de Bourgogne.

## Author Contributions

CGér, NJ, BL and BB designed research studies. CGér, NJ, BL, MT, CGen, MC, and CC conducted experiments and acquired data. CGér, BL, and BB analyzed data and wrote the manuscript. AB, TG, HG, SO, CT, OA, PS, MS, and SA edited the manuscript. FC participated in the experiments perfomed during review process. All authors contributed to the article and approved the submitted version.

## Funding

This study was supported by the Agence Nationale de la Recherche (Labex LipSTIC, ANR-11-LABX-0021), the Ligue contre le Cancer (Comité Grand-Est), the European Regional Development Fund of the Region Bourgogne Franche-Comte (grant No. FC0013440) and by the MiMedI project funded by BPI France (grant No. DOS0060162/00) and by the Région de Bourgogne Franche-Comté.

## Conflict of Interest

The authors declare that the research was conducted in the absence of any commercial or financial relationships that could be construed as a potential conflict of interest.

## Publisher’s Note

All claims expressed in this article are solely those of the authors and do not necessarily represent those of their affiliated organizations, or those of the publisher, the editors and the reviewers. Any product that may be evaluated in this article, or claim that may be made by its manufacturer, is not guaranteed or endorsed by the publisher.
