# Supplementary material for: Human Monocyte-Derived Suppressor Cell Supernatant Induces Immunoregulatory Effects and Mitigates xenoGvHD
Source: Front Immunol. 2022 Mar 8;13:827712. doi: 10.3389/fimmu.2022.827712 (PMC8957111; doi:10.3389/fimmu.2022.827712)
Supplement: Supplementary file 1 [file DataSheet_1.pdf]

Supplementary Figure 1

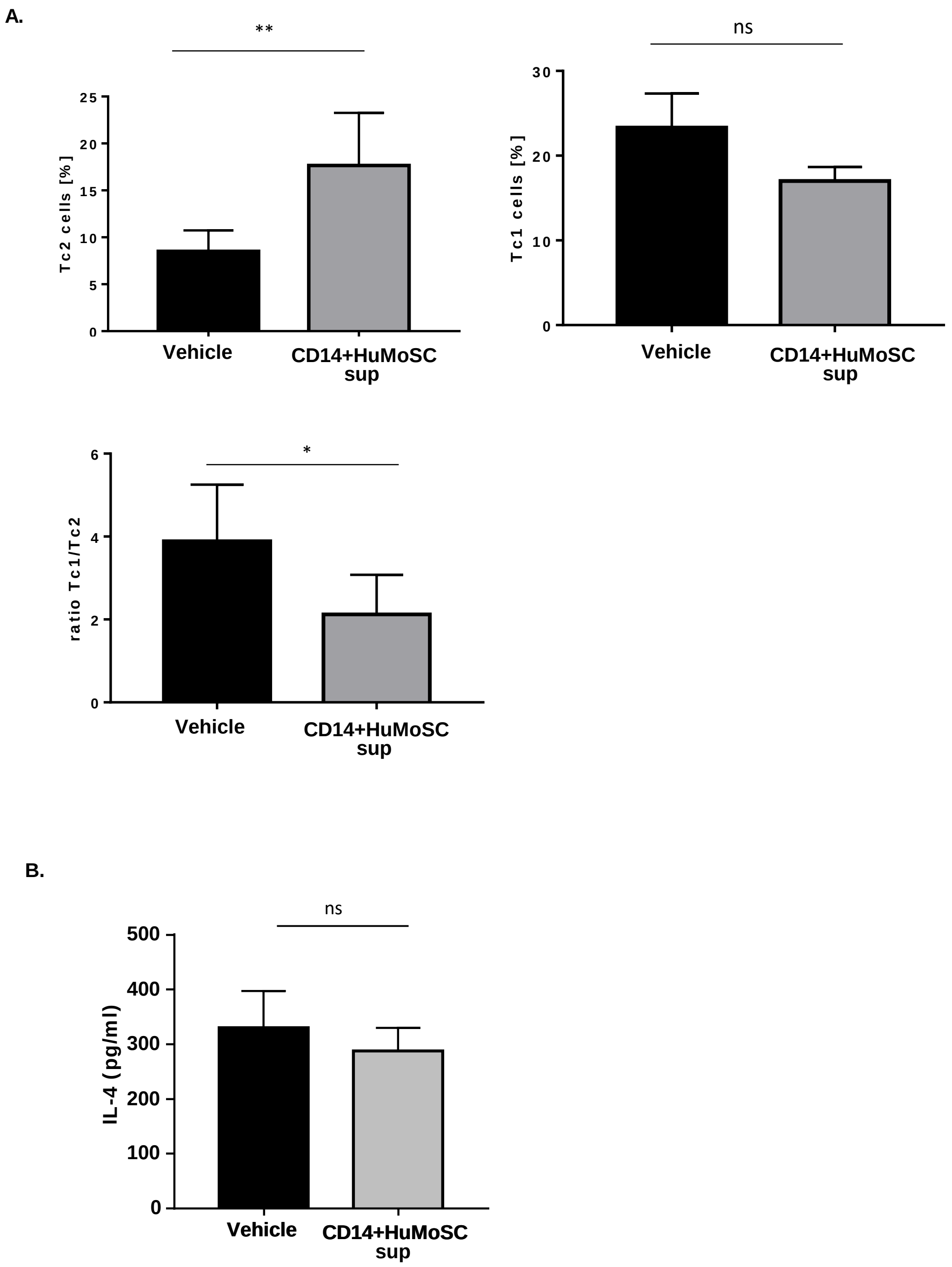

Figure S1. A. After 4 days of stimulation with anti-CD3/CD28 stimulation beads and incubation with either CD14+HuMoSC supernatant or physiological serum (vehicle), T cells were stimulated for 4h with PMA+ionomycine and brefeldin A. A. Cytokine detection among CD8+ cells was performed by flow cytometry after staining with anti IFN-γ and anti IL-4 with BD LSR II and FlowJo software. B. IL-4 concentration was measured in the culture of stimulated T cells treated with or without CD14+HuMoSC supernatant. Results of 3 independent experiments are shown Data are shown as mean ± SEM of representative experiments. Two-tailed Mann Whitney test *: p ≤ 0.05, **: p ≤ 0.01
